# Psychosocial challenges and individual strategies for coping with mental stress among pregnant and postpartum adolescents in Nairobi informal settlements: a qualitative investigation

**DOI:** 10.1186/s12884-021-04128-2

**Published:** 2021-09-28

**Authors:** Caroline W. Wainaina, Estelle Monique Sidze, Beatrice W. Maina, Icoquih Badillo-Amberg, Hazel Odhiambo Anyango, Faith Kathoka, Dorcas Khasowa, Collins E. M. Okoror

**Affiliations:** 1 African Population and Health Research Center (APHRC), Nairobi, Kenya; 2 McGill University, 845 Sherbrooke St W, Montreal, H3A 0G4 Canada; 3 Mum, Baby and Love Trust, Nairobi, Kenya; 4 Mental health consultant, University of Nairobi, Nairobi, Kenya; 5 Department of Obstetrics and Gynaecology, University of Benin Teaching Hospital, Benin, Nigeria

**Keywords:** Mental health, Maternal health, Adolescent, Urban health, Mental stress, Poor settings

## Abstract

**Background:**

This study was part of a project funded under the Grand Challenges Explorations initiative to engage adolescent girls living in the main slums of Nairobi. This involved an innovative co-creation initiative through jointly designing and testing the feasibility of a toolkit of information, skill, and confidence-building, and coping mechanisms that can effectively shield them and their peers against the risks of mental stress during pregnancy and early motherhood.

**Methods:**

Qualitative interviews and discussions from visual methodologies including Photovoice, digital storytelling, and public service announcements were conducted with 30 pregnant and adolescent mothers aged 14–19 years in four informal settlements either pregnant or having a child less than 2 years. The aims included; to generate an inventory of mental stressors during pregnancy and early motherhood; understand how mental stress affects the ability to seek care for themselves and their child, and understand individual coping strategies.

**Results:**

The psychosocial challenges identified in order of importance included: chased from home by the parents; economic hardship; neglect and abandonment by the person responsible for the pregnancy; stigmatization by family, friends, and the community; feelings of shattered dreams; and daily stress related to living in poor and unhygienic conditions. During the pregnancy and early motherhood, the participants experienced feelings of embarrassment, shame, hopelessness, and to the extreme, suicidal thoughts clouded their minds. Main coping strategies included social isolation for some, socializing with other pregnant and adolescent mothers, and negative behaviors like the uptake of illicit drugs and alcohol and risky sexual relationships.

**Conclusion:**

The unpreparedness for early motherhood infused with inadequate psychosocial support led to increased mental stress and risk of depression. The interconnection between the triggers to mental stress showed the need to focus on a multifaceted approach to address the wellbeing of pregnant and adolescent mothers.

**Supplementary Information:**

The online version contains supplementary material available at 10.1186/s12884-021-04128-2.

## Background

Globally, the population of adolescents has continued to rise over the past decades. According to the 2017 UN World Population Prospects, 14% of the world’s population live in sub-Saharan Africa (SSA), of which 23% are adolescents aged 15–19 years [1]. Adolescence is a critical developmental period where sexual and reproductive behaviors and attitudes, with life-long implications on health and wellbeing, begin to emerge. Adolescents living in SSA are at a heightened risk of poor reproductive health outcomes including early and unwanted childbearing associated with low educational attainment, poverty, gender inequality, and social norms which in turn limits adolescents’ access to sexual and reproductive health (SRH) information and services [2]. Early pregnancies and childbearing lead to major implications for the well-being and welfare of adolescents, including mental wellbeing.

### The plight of women including adolescents living in urban poor environments

An important dimension of Africa’s demographic system is the phenomenon of high levels of urbanization, fueled mainly by persistent migration from rural areas in search of better livelihood opportunities. The urban population in the region is set to increase from the current 37% to over 60% by 2050 and the high rate of urbanization has been linked to the increasing urbanization of poverty, with approximately 70% of all urban residents in the region estimated to be living in slums or slum-like environments [3, 4]. The growth of slums, characterized by overcrowding, social and economic marginalization, poor environmental conditions, insecurity, and little or no basic social services, has intensified in sub-Saharan Africa. Researchers have shown that some urban health and social indicators have either deteriorated appreciably or even reversed in favor of rural areas [3, 5]. In particular, the new face of urban poverty has been linked to adverse sexual and reproductive health outcomes for the urban poor, which include high rates of unwanted pregnancies, higher fertility, sexually transmitted infections (STI’s), and poor maternal and child health outcomes. The Nairobi informal settlements typify sub-Saharan Africa’s urban crisis [6–11]. Living in urban informal settlements has been associated with limited ability for women to control and implement their fertility preferences. Compared to other urban and rural areas, women in urban informal settlements have reported high rates of unintended pregnancies [12] and non-use of contraceptives among post-partum women. For instance, in a study conducted in Korogocho and Viwandani slums in Nairobi, about a quarter (28%) of postpartum women, at risk of another pregnancy, were not using a contraceptive method [12–14]. Additionally, most of the public health facilities in Nairobi informal settlements do not have skilled personnel or the basic equipment needed to provide quality information and essential health services [8]. Other challenges in the provision of healthcare services include but not limited to inadequate maternal and child health (MCH) services to engage pregnant/parenting adolescents; lack of preventive or treatment mental health programs in health facilities, and lack of programmatic focus on parenting and or mental health in the training of healthcare staff [15]. In facilities that provide the basic MCH services, perinatal mental health is usually not prioritized due to other competing health challenges that require urgent attention ([16, 17]). Therefore, the mental health and wellbeing of slum populations, and adolescents, in particular, are constantly under threat.

### Why focus on both the physical and mental wellness of pregnant and postpartum adolescents?

It is estimated that 11% of all births worldwide are among adolescents 15–19 years of age with the majority occurring in low resource settings [18]. The sub-Saharan African region continues to experience the highest rate of adolescent births worldwide, with approximately 120 births per 1000 adolescent women [19]. Adolescent pregnancies are a major risk factor for mortality and morbidity [20]. Available evidence also suggests that adolescent girls are at heightened risk of mental health stress since their pregnancies are often unwanted and compounded by risk factors such as lack of social support, stigma, intimate partner abuse, and economic hardship [21] as well as testing positive to HIV [22]. Lack of proper preparation and essential information (infant feeding and caregiving practices and prevention of childhood illnesses) provide the basis for increased mental stress in parenting adolescents [15]. Despite the urgent need for adolescent mental health resources, psychiatric hospitals remain the dominant mental health resource in urban settings and are not utilized due to a high degree of stigma associated with mental health [18]. Data gathered from the Mental Health Atlas project reports that 40% of African states have no allocated budget for mental health and 20% do not have mental health legislation [18]. Challenges to the provision of mental healthcare include lack of mental health specialists; lack of prescribing guidelines; limited evidence on feasible detection and treatment strategies for maternal mental disorders; and stigmatizing attitudes among primary healthcare staff and the communities [23].

## Study objectives

This study was part of a bigger project that aimed to engage pregnant and parenting adolescents living in the main slums of Nairobi. The engagement involved an innovative co-creation initiative through jointly designing and testing the feasibility of a toolkit of information, skill, and confidence-building and coping mechanisms that can effectively shield them and their peers against the risks of mental stress during pregnancy and early motherhood.. The project engaged pregnant and adolescent mothers living in four informal settlements of Nairobi (Korogocho, Viwandani, Kangemi, and Kawangware). The research team carried out the project in three (3) consecutive phases: *Phase 1* consisted of facilitated discussions with a sample of adolescent girls aged 12–19 years old, pregnant, or with a child less than 2 years. *Phase 2* aimed to develop a toolkit to shield adolescent mothers from stressors and *Phase 3* tested the effectiveness of the designed toolkit in promoting resilience and stronger minds among adolescent mothers living in slums.

This paper reports on findings from Phase 1 of the project and the main objective is to describe the triggers to mental stress identified by adolescents and their coping strategies in the informal settlements of Nairobi, Kenya.

## Methods

### Recruitment and field procedures

The project team worked with community-based organizations that currently engaged the pregnant adolescent mothers in each of the slums, to identify and recruit potential participants’. The CBOs provided mentorship, connected them to informal jobs, and or empowered them through skills training. To obtain a sample of girls with a large array of experiences with stress, the project team screened all eligible girls using the validated Edinburgh Postnatal Depression Scale (EPDS) tool that has been adapted and used in the Kenyan context [15]. The EPDS scale measures the level of depression categorized either as normal (0–7), mild (8–12), moderate (13–19), and severe (20–30). The girls exhibiting a depression score ≥ of 13 and above at the screening stage, were to be excluded from the study and referred for clinical evaluation but fortunately, we did not get any that ranged above a score of 13 from those recruited. In total, the research team recruited 30 adolescents and engaged them in participatory discussions between November and December 2019. Table 1 below outlines the demographic characteristics of the girls recruited.

**Table 1:** List of participants in the different study sites and their ages

List of participants for Sasa Mama Teen project							
Korogocho		Viwandani		Kangemi		Kawangware	
1	5 months pregnant, 16 years	1	5 months baby, 17 years	1	Pregnant, 2 months, 15 years	1	1. 5 years child, 19 years
2	5 months pregnant,	2	6 months child, 17 years	2	pregnant, 7 mnths, 16 years	2	10 months child, 17 years
3	7 months pregnant, 17 years	3	7 months, 18 years	3	4 months pregnant, 16 years	3	2 months pregnant, 16 years
4	6 months pregnant, 18 years	4	9 months child, 16 years	4	Pregnant, 1 month, 17 years	4	1.5 years child, 17 years
5	6 mnths child, 18 years	5	5 months pregnant, 17 years	5	1 months pregnant, 15 years	5	1 year child 15 years
6	18 months child, 18 years			6	9-month child, 17 years	6	9 months child, 17 years
7	3 months pregnant, 15 years			7	Child, 2 months, 17 years	7	6 months child, 18 years
8	1-year child, 17 years			8	Pregnant, 8 months, 17 years	8	14 months child, 17 years
9	3 weeks pregnant, 15 years						

The study used visual methodologies (Photovoice, digital storytelling, and public service announcement), qualitative interviews, and focus group discussions to explore lived experiences with pregnancy and motherhood.

Photovoice is a collaborative participatory methodology that allows participantsto use their photographic work to share their lived experiences and to reflect on the triggers to mental stress and their strengths that have enabled them to cope [24]. This approach is well suited for capturing the experiences, needs, and agency of mothers living in informal settlements. The project team received a three-day training on visual methodologies by a McGill lecturer and expert on the visual methodologies before commencing fieldwork. The research team met with the 30 participants and introduced the project including the research objectives and the methods of engagement proposed during the first workshop. The research team divided the participants into four groups based on their study site, (Korogocho 8, Viwandani 6, Kangemi 8, and Kawangware 8) and introduced each group to the photovoice concept and the ethics applied. The ethics dwelt on how to minimize risks and harm to both the participants and the people they will photograph. The team then trained the adolescents’ on-camera use and photo ethics (no face, no identification). The phrase “no face” meant that the photos taken should not be of a person’s face or anything that identifies the person or area. After the training, each adolescent received a camera to take up to three photos in their communities and a prompt question or initial theme to give them focus, in this case, *“what is your experience with pregnancy and motherhood? what do you consider as causes of mental stress? How do you cope when stressed*? *”*. The research team hired a few young men to provide security for the participants during data collection. The data collection took approximately 2 h. Photos taken were then reviewed to ensure they met the ethical requirements (photos that identified individuals or had other identifiers were removed). The participants then discussed the one photo to keep and print based on the prompt and adhering to Photo ethics. The research team discussed with the participants on the selected photos. Through the guidance of the SHoWED principle (**W**hat do you **S**ee here, what is **H**appening here, how does this relate to **O**ur lives, why does this concern **E**xist, what can we **D**o about it) [24], each participant was able to identify the photos that communicated her perceptions triggers of mental stress, coping strategies as well as potential solutions. For the captions, the participants captioned the photos using what they perceived the photos communicated. The discussions were audio-recorded and the recording transcribed directly into English by a bilingual transcriptionist. The research team used the description of the photos provided by the participants to caption the photos in this paper. To note that process photos of the participants taking the photos were also collected. This provided proof that the participants were the ones who had taken the photos.

Digital storytelling is a visual participatory method that involves video narration of a person’s lived experience. It is a creative process that engages the participants in a dialogue about their lived experiences and conveys emotive and thought-provoking messages [25]. In digital storytelling, participants are encouraged to share their experiences within a story circle group and once every group member has shared their story, the different groups decide on a common story that is relevant and applies to all [26]. In our engagement, the 30 participants used digital storytelling to develop a storyboard on their lived experiences. A storyboard comprised of the different aspects of their lives and who in their life participated in that aspect of the story. The board gave the story flow and enabled the participants to work together and agree on the different aspects of the story. The participants from each study area developed a story from a common shared theme. One team member provided narration for the story while another team member took photos of the other team members dramatizing the parts of the story. The team used a free video editing platform called wevideo.com to develop the videos. The videos comprised of the photos taken dramatizing the aspects of the story, the audio narration, and other background effects including the sound of a child crying clouds to depict transition to another day, and other photos from their environment that added some reality to the story, for example, a dirty river, or a closed house. Discussions then followed on the theme chosen for the video and its relation to mental stressors as well as potential solutions. The discussions were audio-recorded and the recording was directly transcribed into English by a bilingual transcriptionist.

The 30 adolescents made separate short video clips with messaging that they considered as potential solutions to the challenges they faced including telling boys/men to use condoms and or let them study if they are not willing to accept the pregnancy and responsibilities of raising children. The participants presented the video clips as public service announcements and these complemented the digital storytelling and photovoice discussions. The research team held another workshop with the 30 participants and shared the findings received by each group. The different groups then shared if the findings applied to their group as well. A rich discussion ensued from the findings by the different groups including validation of some of the findings being similar across the different study sites.

## Ethical considerations

The research team took several measures to minimize any potential stress on respondents. The team carried out all activities in a suitable environment selected within the communities where the girls lived and that ensured a safe space for discussions. The respondents received information about the purpose of the study, the use of the data before seeking their written consent to participate in the study, and about the study benefits and their rights as study respondents. As all adolescents selected for this study were pregnant or recent mothers and considered as emancipated minors, an adult consent process was used [27]. The research team de-identified all the transcripts before analysis to ensure the privacy of respondents. All respondents received transport/time reimbursement for their participation in the study. The study received ethical approval from the AMREF Health Africa Ethical and Scientific Research Committee (P532/2018). The study also applied for registration with the National Commission for Science, Technology, and Innovation.

## Data analysis

The research team used the interview guide and initial transcripts to identify main themes and sub-themes. This informed the codebook development which the project team comprising of the principal investigator, and a qualitative researcher, and a junior researcher used to code the rest of the transcripts. Th same process was used for the focus group discussions and the in-depth interviews. All the coded data were further classified and analyzed through queries and word trees in *Nvivo Q10* Software using thematic content analysis [28].

## Findings

The findings below are categorized as per the research objectives of the project:

1. Triggers to mental stress during pregnancy and early motherhood

### Psychosocial challenges experienced

#### Being chased from home

**Fig. 1:**
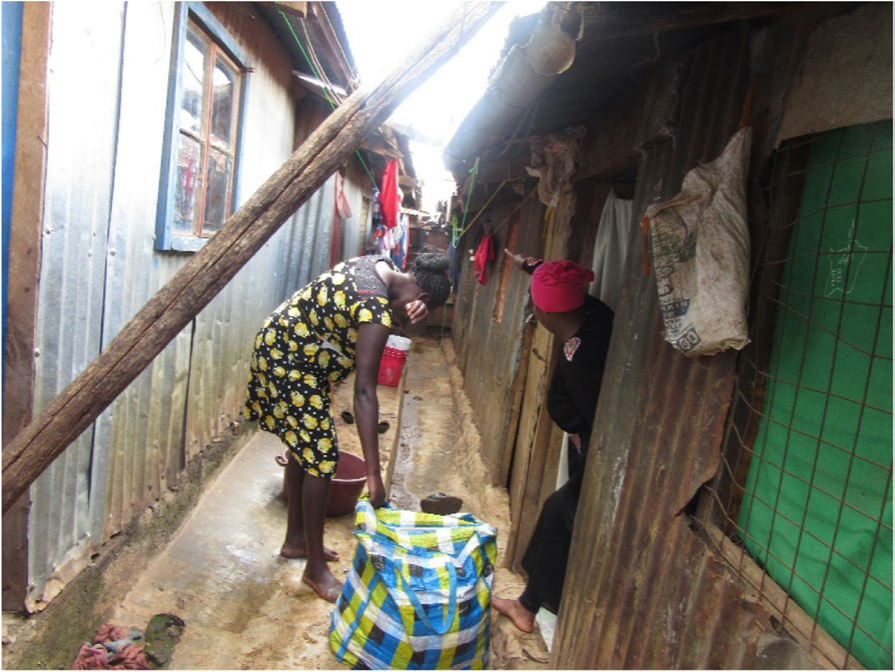
Chased from Home by the family once the pregnancy becomes evident

This was a common occurrence in all the informal settlements with parents, especially the mother, angered, and embarrassed by the girl for getting pregnant while in school. Figure 1 below captures a mother chasing away a pregnant adolescent from home after realizing she was pregnant. The parents and guardians viewed a pregnant girl as having wasted their (parent’s) time and money especially for those families that struggled economically to meet household needs. Because of the feelings of disappointment, embarrassment, and unnecessary burden, the parents and or guardians chased the pregnant adolescents back to the person responsible for the pregnancy. To restore some dignity, some parents opted for the girls to go through an abortion, which led to more stress for the girls, as they all knew the adverse effects of abortion. Some of the participants chose to run away from home to avoid forceful abortions, while others stood their ground and opted to face the consequences of being mistreated by their families. In a desperate attempt to find support, adolescents found companionship in negative friendships that introduced them to risky behaviors like alcohol, drug abuse, and prostitution.

*I thought of abortion and then I thought no, this could be the only child in my entire life. So I kept it and gave birth. I thought of committing suicide … I thought of many things, I told my aunt and my mother. Both of them didn’t want to see me because I was pregnant. They used to tell me I went to get pregnant while being in school; I should know how to deal with it. They said a lot of hurting words, you should terminate it, but I felt no … I should only put up with all this (IDI, Kawangware)*

*They (parents) claim that they never had babies out of wedlock. So, it is an embarrassment to them for you to have a baby out of wedlock and they also claim that our age mates are still in school (FGD, Kangemi)*

#### Economic hardship

**Fig. 2:**
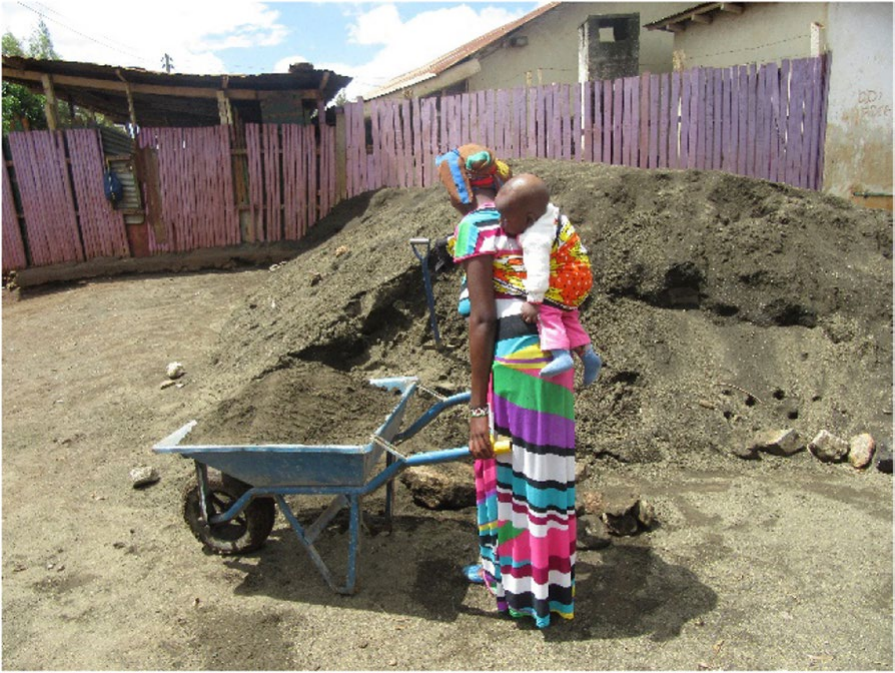
Captures the challenge of being forced to look for work with the baby due to the inability to afford daycare

Girls needed financial support to afford basic needs like food, shelter, and clothing for themselves and the baby, cater to hospital fees including delivery, treatment, and tests. With little or no source of income as well as limited financial support from their families, the inability to meet the needs presented as triggers to stress for the adolescents. To deal with the economic challenges they opted to look for odd jobs to support themselves and the baby. Getting employment came with its challenges; the participants indicated being unable to afford daycare while they look for work, and other times being denied opportunities to work because of carrying their babies to seek work. In some instances, they did get jobs with the condition that the baby would not interfere with the work assigned to them. Figure 2 below shows photographic evidence of this challenge; some were not age-eligible for employment while others had no academic qualification since they dropped out of school.

*I was stressed because of the pregnancy and didn’t have money to cater for my baby’s expenses … my mum used to give me food, but was not helping as before … was in deep thoughts because my partner rejected me … when I borrow money from him, he just stares at me … I decided enough is enough and opted for casual jobs to take care of my baby (IDI Adolescent mother)*

*After I gave birth, I got stressed because I didn’t have somewhere to leave my baby as I go to work, others at home are working and I don’t have money to pay for baby care…so I used to work with my baby on my back (IDI, Viwandani)*

#### Neglect/abandonment

**Fig. 3:**
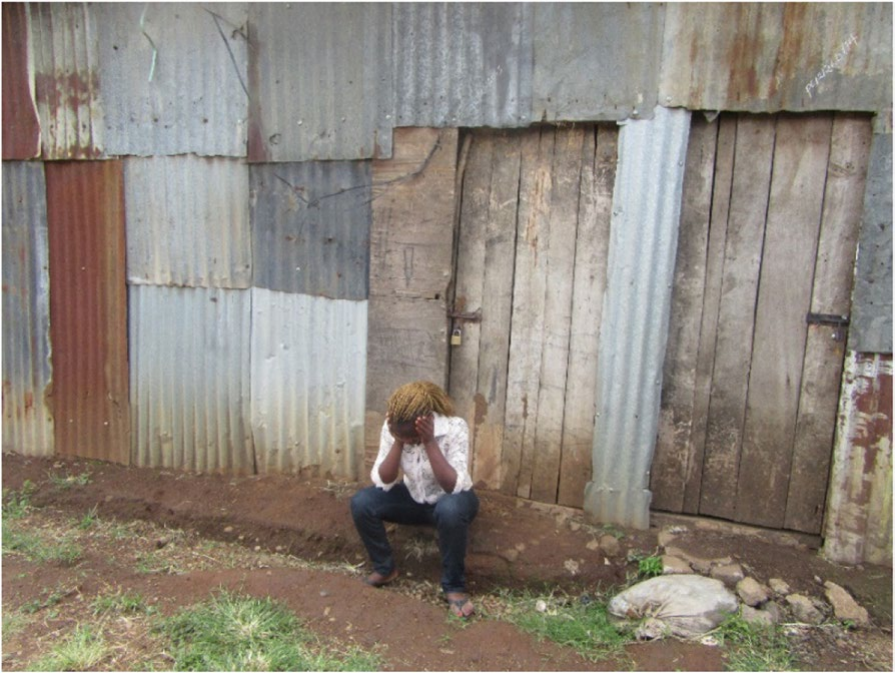
Neglect and abandonment by family and friends

Adolescents faced the challenge of neglect or emotional abuse by their partners or the person responsible for their pregnancy. The men either denied responsibility or accepted but offered no support. This abandonment led to girls contemplating abortions as they felt helpless and others indicated thinking of committing suicide. Figure 3 below shows a pregnant adolescent girl extremely stressed and in deep thought after the boyfriend denied her pregnancy. In some cases, the adolescents moved in with the partners to shield themselves from such feelings but ended up more stressed as the men offered no support to the adolescents.

*He denied claiming it was my mistake and he was not responsible because I didn't play safe … I did not understand what he meant but told him if he was not taking responsibility, I will have an abortion … he told me to have an abortion because he can’t be a father at that age (IDI Kangemi)*

*He accepted because he saw the baby resembles him so he can’t deny it. So I moved in to live with him, little did I know I was coming to suffer. For example, if he works and gets KSh 200, instead of telling me to get this one hundred and buy pampers for the baby, he just goes and drinks alcohol and chews Miraa (illicit drug), but I just understand because I accepted to get married. I just persevere and wait for God to see if he will change (IDI, Kawangware).*

#### Stigmatization by family, friends, and community

**Fig. 4:**
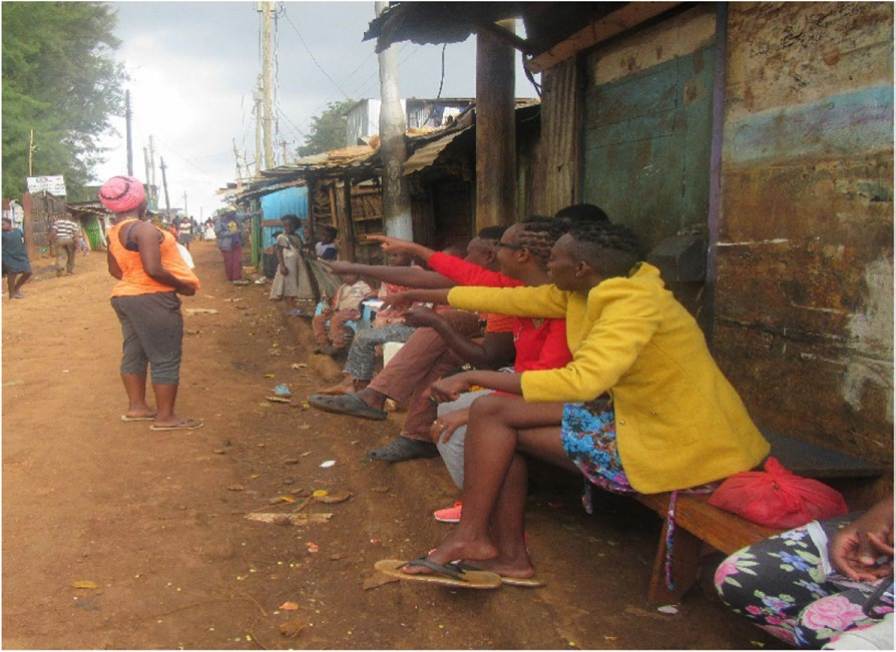
Stigmatization by friends

Participants encountered a lot of backlash from family, friends, and the community. They were insulted, gossiped, pointed at, and discussed as portrayed in Fig. 4 below. They were unable to continue with school due to stigma from schoolmates and teachers. Feelings of discrimination at the health facilities by healthcare staff and other patients made seeking healthcare difficult for most of the participants during the pregnancy period. The stigmatization experienced left the participants feeling isolated, judged, ashamed, and unworthy.

*I was in school, then one day I was sent by my teacher to take a certain book to the school nurse. So while seated there with the nurse, she became suspicious of me and requested if it was ok to conduct a pregnancy test which I accepted. After the test was done she never gave me the results but instead, she told the school matron who in return told her daughter about my pregnancy test. I, therefore, discovered when I heard rumors from other students that I was pregnant (FGD, Kangemi)*

*My friends are gossiping about me up to date. Neighbors gossiped about me saying that taking care of the baby is not an easy job and I will not manage it at my age (IDI, Kawangware).*

#### Feelings of shattered dreams

Some of the girls indicated how being pregnant or a mother at such an age had robbed them of educational and career opportunities, opportunities to be adolescents, freedom of movement, freedom to have fun, buy things for themselves, have friends. Such feelings affected how they perceived the pregnancy and motherhood journey.

*… this lady with a baby has to hustle, she could be in school, the other is working for her children … if she didn’t have she would be able to take care of herself with ease. Today I can go and spend a night in a friend’s place but when you have children, you have to provide a home for them … someone can buy you food, maybe because they love you, but when you are with a baby, no man can even whistle at you (FGD Photovoice)*

#### Daily challenges with living in a slum environment

**Fig. 5:**
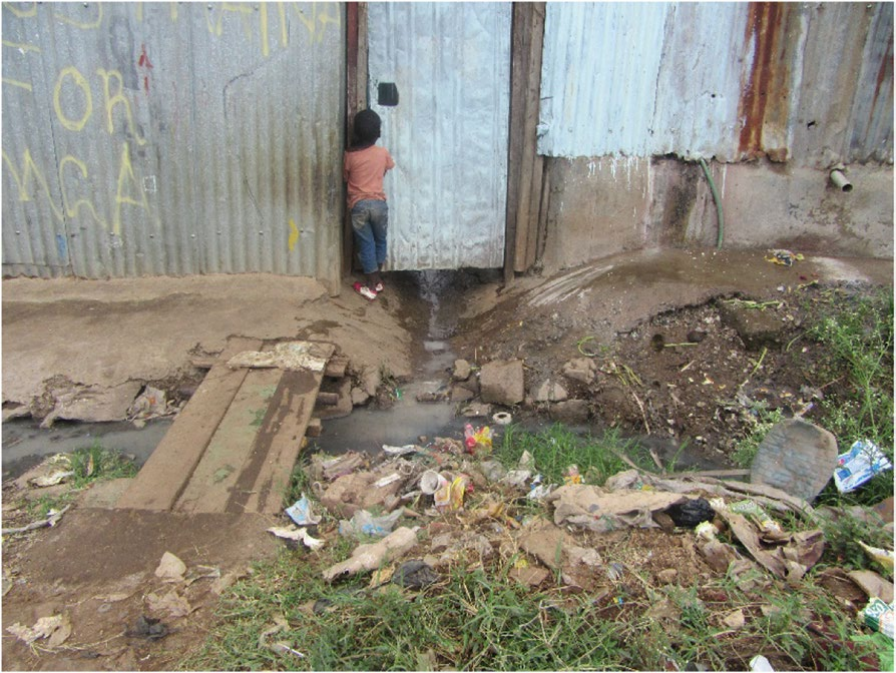
Photo captures the dirty environment that the mothers and their children live in

Living in the slums has challenges like unhygienic environments, open sewers, fecal waste, and garbage as shown in Fig. 5 below. This affects the urban slum dwellers in exposing them to various acute infections. This impact on the adolescent mothers was hugely felt as they grappled with how to take care of themselves in this environment and their babies. According to the girls, the event of sickness because of the unclean environment resulted in high amounts of stress, due to the baby being sick and not knowing how to take care of the sick baby, the lack of financial resources to take the baby to a hospital only adding more stress to the adolescent girl.

*The dirty environment is a challenge … there are trenches all over and you find may be the child went to play there with dirty water … dirty environment causes diseases to the child, the baby gets sick and you don’t have money to take the baby to hospital … this stresses you as you don’t have money for the hospital (FGD, Viwandani)*

2) Understand how stress affects their ability to seek care for their pregnancies or young infants (findings obtained from the qualitative interviews with the participants)

The main challenge was a lack of support. This included support from family, friends, school, and the community. Some considered support as financial and indicated not receiving any support or inadequate support. The adolescents mentioned the reasons for lack of financial support as their families’ socio-economic status where most lived in abject poverty, surviving on a meager income, having competing interests, or not willing to support as punishment for the adolescent getting pregnant.

*My mum is the only support but now she cannot help me because she is the one paying house rent. After all, since my dad got into an accident … .he depends on my mum who also pays school fees for my sister who is in form two (IDI, Kangemi)*

*I get help but not as much as I want, at times am given food, other times being told to go and look for food … I share with my mother and friends when I have a problem and they sort me out … but sometimes they will say they do not have money, my mum can sometimes give me or she can refuse (IDI, Korogocho)*

The adolescents mentioned a lack of emotional support especially from family members after discovering they were pregnant which exacerbated the stress the adolescent girls were already experiencing. While the girls mainly relied on their mother for emotional support during pregnancy, the mother’s reaction to the pregnancy determined how the girl handled the pregnancy and subsequent motherhood.

*I wanted to terminate my pregnancy because I had no one to support me. I was also stressed up because my father warned me not to go back home, the baby’s father denied being responsible and my employer chased me away…my father warned me not to go home as my stepmother did not want to see me … no one supports me when I am stressed up (IDI, Kawangware)*

The participants mentioned social support as a challenge that influenced the feelings they had towards their pregnancy and subsequent motherhood. Friends and community provided inadequate social support. The adolescents felt that some of their friends were unsupportive by gossiping or laughing about their pregnancy and early motherhood situation. This made it impossible for them to hang around their friends. The community on the other hand felt the adolescents were a bad influence on their children and thus kept them away. This led to the adolescents feeling isolated socially and increased their incidences of stress.

*No one supports me … because even my friends are not trustworthy. When you tell your issues, they end up gossiping about you (IDI, Kawangware)*

*Some of my relatives wanted me to abort, they took me to the police station because I had refused to say who is responsible for my pregnancy, but I said. I refused to abort. The baby’s father was ready to pay for an abortion. People used to call me names. I used to cry my friends used to gossip too bad about me...they rejected me and separated themselves from me (IDI, Viwandani)*.

The participants mentioned healthcare support as a challenge. They indicated that the staff were normally harsh towards them and treated them unfairly, blaming them for getting pregnant at an early age. Some of the participants indicated not receiving healthcare information such as infant feeding and childcare giving practices in health facilities. Most of the participants indicated opting not to visit such health facilities instead of going to further facilities where the staff treatment was better.

*The treatment by the staff is a problem. They disrespect you and can even insult you just because you are pregnant at this age. They make you feel you have committed the biggest mistake (FGD participant, 2*^*nd*^*Workshop).*

The support that the adolescents wanted to see in their lives and the lives of other adolescents in similar situations included financial support to start businesses, to go back to school, or vocational training. They also indicated that receiving information on birth preparedness, problem-solving, and confidence-building skills would be of great support for adolescent mothers in how they should take care of themselves and their children/babies.

*I just want to go back to school (insisting) and then I get someone who is supporting me. You see, someone paying for my school fees and giving me these small things. You know these small things are the ones that make someone get pregnant, things like sanitary towels, body lotion, but mostly pads (IDI, Kangemi)*

*It (problem-solving skills) will help adolescent mothers to know how to approach their parents because most of them run away from home as soon as they realize that they are pregnant … it will help avoid/reduce abortion cases (IDI, Kawangware)*

3) Understand individual experiences for coping with stress (findings obtained from qualitative interviews with the participants)

#### Social isolation

To cope with stress caused by stigmatization, neglect from family and friends, and their negative feelings and experiences towards pregnancy and motherhood, a few of the adolescent mothers chose to isolate themselves from other people. Apart from trying to avoid people, the girls mentioned sleep as a coping strategy when faced with stress.

*I do engage myself in work or sleep. I don’t tell people about my problems (IDI, Kangemi) …*

*I try and avoid people and only share with my friend (IDI, Korogocho) … I distance myself from people when I feel like I don’t want to associate with them (IDI, Viwandani) … I don’t share my issues with anyone else since my friend moved to Mombasa. I only pray for God to help me (IDI, Kawangware). Myself, I just sleep. I can go out and see things that will make me cry, so I sleep (IDI, Kangemi)*

#### Spending time with friends in similar situations

Some adolescent mothers indicated that during the points of stress, they would spend time with their friends, some new ones; others preferred spending time with other adolescent mothers who could encourage them and guide them in how to take care of their pregnancy or baby. Because of their pregnancy, some of the adolescents had lost most of the friends they had, optionally or due to stigmatization, and only retained a few that they trusted or that encouraged them.

*I do talk to my friends although some can't help you because they don't understand my situation. I like meeting up with friends who give me hope … like one of my friends, who is someone who has already given birth... she also told me not to overstress myself because it can cause miscarriage … I have a friend who is pregnant too. She encourages me and tells me to stay strong. IDI, Korogocho).*

#### Taking up other habits/hobbies

The adolescent girls looked for different avenues to release their stress. Some included reading books, watching movies, and spiritual hobbies like praying. Others were drawn into drugs and hanging out with ‘dangerous groups’. The participants indicated that idleness brought about by lack of jobs and dropping out of school, and being chased/running away from home led to some participants joining bad company or going into drugs. Drugs such as Miraa were an easier option to deal with stress faced by the adolescent mothers. The girls mentioned its increasing use in two informal settlements regardless of the harm it may cause to the mother and the baby.

***M*** *: Does stress make adolescent mothers get involved in drug abuse such as the use of Miraa (asked about a photo of a girl chewing Miraa)?Yes (all respondents)* ***M*** *: Is the use of Miraa a common practice in pregnant adolescent mothers in this area? Yes (all respondents)* ***M:*** *How does it make them feel? It makes them feel happy … It reduces stresses (FGD, Photovoice, Kangemi)*

*Most adolescent mothers have no jobs to keep them busy, thus they get into drug abuse, theft, joining the bad company, gossiping (FGD, Photovoice, Kangemi) Idleness makes one enter into many risks, drugs, pregnancy, of which I put myself in. taking drugs is one way of reducing stress but unsafe way … they know it’s bad but what they know is that it reduces stress (FGD, Photovoice, Kawangware).*

## Discussion

The findings from the engagement with the pregnant and adolescent mothers provided insight into the triggers that lead to mental stress among adolescent mothers and the different ways in which they coped with the stress. The themes captured through the engagement including abandonment, stigmatization, neglect, feelings of shattered dreams, and overall lack of adequate support, are in line with findings from other studies conducted in similar settings and among the same age group [15, 29, 30].

This paper also highlights the interconnections between the different triggers and their effect on the mental wellbeing of adolescent mothers. Being chased or running away from home, neglect and stigmatization led to economic challenges including the girls being unable to provide the basic needs for themselves and their young children. The stress due to lack of financial ability was connected to poverty derived environment where they lived, in their inability to afford healthcare expenses in the instances of sickness, a preserve of the disadvantaged, and people who live in informal settlements [31]. This compounded by challenges they experienced in accessing and utilizing youth-friendly healthcare services, put the adolescent mothers at a great disadvantage in regards to their health. This finding is supported by another study on adolescent pregnancies and challenges that highlights inadequate healthcare support accorded to pregnant adolescents in such contexts [15].

Locked in the seemingly unending cycle of poverty, dropping out of school provided a little option for the girls but to look for odd jobs to cater to their basic needs. This inadvertently propagated the girls into a self-reliance mechanism to ensure that they have what they and the children need. Even though all the girls indicated a desire for a better future for their children, unfortunately, research shows that children of mothers with less education are likely to be uneducated [32].

Support defined by the girls as economic, emotional, and social was inadequate. Lack of adequate economical/financial support important for providing the basic needs including food, shelter, and healthcare caused the girls to feel neglected and exposed them to feelings of fear, despair, worthlessness, embarrassment, and helplessness leading to ideations of abortion and even suicide to flood their minds. Adolescent girls interviewed in another study conducted in the informal settlements of Nairobi share the same sentiments on feelings of shame, embarrassment, fear as a result of the unintended pregnancy [12].

Stigmatization by family, friends, and community stood out strongly as a factor that exacerbated the mental stress the girls were going through and constrained access to social support. The need to belong and accepted was challenged by the societal and cultural norms that depicted such girls as wayward, bad mannered, too young to get pregnant, and spoilt. According to other related studies on adolescents, stigmatization increased the vulnerability of the girls to risky health and sexual behaviors including increased sexual occurrences with different partners, increased contraceptive use, and use of sex for financial gain (prostitution). Other studies show that depressive symptoms that emanate from factors like stigmatization impede the decision-making process, making adolescents more susceptible to high-risk practices and behaviors [14, 22].

The participants derived the coping strategies from their experiences and depended largely on the support they received regardless of how small it was. Unplanned, unintended pregnancies for such age needed different coping mechanisms to overcome the helpless state that the girls felt. Resilience powered their determination to survive and take care of their children, the feeling of making it as a mother, and having a healthy child was a motivator and a positive coping mechanism. Taking up new habits, making new friends in similar situations replaced the feelings of stigmatization and neglect and build self-confidence and reduce the impact of the stress the girls were facing. On the adverse side, some of the new habits/coping mechanisms like early marriages, negative behaviors, seemed not to help in reducing mental stress but more so exposing the adolescents to adverse outcomes including gender-based violence, sexual exploitation, and more unplanned pregnancies. A qualitative study among adolescents with HIV in Uganda described the earlier coping strategies as problem-focused strategies concentrating on how to manage the problem, while the latter as emotion-focused strategies concentrating more on avoidance and distraction [33]. Beguy et al. in their study refer to the two as protective and risk factors, protective being those habits/behaviors that motivate a positive attitude towards entry to motherhood. Risk (model and vulnerability) factors are those that propagate the adolescents towards feelings, habits/behaviors that reflect the despair, low self-esteem, perceived low life chances [34].

Small capital to start a business, affordable quality daycare options, paying off school fees for those who wanted to return to school, vocational training for skills development all represented their desire for a better future for themselves and their children. Problem-solving skills, confidence building, maternal and child healthcare information were mentioned as needful in supporting the adolescents deal with the stresses they were undergoing including the relationships with their families. Engagement of families in understanding how to support pregnant and adolescent mothers was suggested and according to a study conducted on HIV adolescents employing a family-centered intervention was shown to achieve sustained behavior change [35].

## Conclusion

Psychosocial support for pregnant and postpartum adolescent girls living in poor urban settlements is critical. Maternal depression increases among adolescent mothers who are more prone due to various reasons, with the major one being, they are not ready for motherhood. Support offered and or interventions implemented in urban poor settings target mainly financial opportunities and physical support. Inadequate support reported or interventions implemented to offer psychological/emotional support. This paper aims to contribute to the growing attention on teenage pregnancies in Kenya and globally by providing an insight into the lived experiences of pregnant and adolescent mothers and highlighting factors to be considered in supporting them to live more resilient and healthier lives and ultimately reduce the number of recurrent pregnancies from this age group.

## Supplementary Information

**Additional file 1.**

## Data Availability

Data from the study is available to be shared upon request to the corresponding author. This will be in the condition of anonymization to protect the identity of the study participants.
