# Supplementary material for: Psychosocial challenges and individual strategies for coping with mental stress among pregnant and postpartum adolescents in Nairobi informal settlements: a qualitative investigation
Source: BMC Pregnancy Childbirth. 2021 Sep 28;21:661. doi: 10.1186/s12884-021-04128-2 (PMC8480022; doi:10.1186/s12884-021-04128-2)
Supplement: Supplementary file 1 — Additional file 1. [file 12884_2021_4128_MOESM1_ESM.docx]

**Psychosocial challenges and individual strategies for coping with mental stress among pregnant and postpartum adolescents in Nairobi informal settlements. A qualitative investigation**

Caroline W Wainaina, Estelle Monique Sidze, Beatrice W. Maina, Icoquih Badillo-Amberg, Hazel Odhiambo Anyango, Faith Kathoka, Dorcas Khasowa, and Collins E. M. Okoror

**In-depth interview guide for adolescent mothers – Phase 1**

**Socio-Demographic information**

| Age (Years) |  |
| --- | --- |
| Sex: Male/ Female |  |
| Highest level of education attained | 1. No education 2. Pre-primary 3. Primary 4. Post-Primary/Vocational 5. Secondary/'A' Level 6. College (Middle Level) 7. University |
| Marital status | 1. Never married/Single/in a relationship 2. Never married/ Single/Not in a relationship 3. Married 4. Divorced/separated 5. Widowed 6. Don’t know 7. No response |
| Currently living with the spouse/partner | Yes  No  Other |
| Number of children |  |
| Current employment status | 1. Unemployed 2. Formal employment 3. Casual 4. Farmer 5. Business 6. Student 7. Other (specify): ____________________ 8. Does not know 9. No response |

| **Icebreaker questions**  So (Name of respondent),   - How are you today? - How are things at home?   *For pregnant participants ONLY*: How is your baby doing? Is it your first pregnancy (if no: how many other children do you have? What are their ages? How is the motherhood journey so far in general?  **Pregnancy and Motherhood experiences and challenges**   1. Please share with me how you felt when you received the news that you were pregnant with this child/your last child?  - In terms of how you reacted; did you feel the discovery was positive or negative? - In terms of how the baby’s father reacted - In term of how your parents, friends, school teachers, and neighbors reacted  1. Did you go to see/have you seen a service provider during the pregnancy for antenatal or postnatal care? If yes, please tell me which facility you visited (to factor in the different visits) 2. Please tell describe the reasons for why you chose this facility for the antenatal, delivery and or postnatal services (to start review of tools from tomorrow)   Probe:   - - Affordability, am able to afford or its free   - Distance, nearer or far from me   - Preferred by people in my age group   - Provide good services, friendly staff   - They have support for young mothers like me (support groups, health education)   1. They give good information  1. Please tell me if you have experienced challenges in attending and accessing antenatal, delivery and postnatal services.    1. Affordability, not able to afford    2. Distance, nearer or far from me    3. Rude staff who discriminate against me as a pregnant or adolescent mother    4. No follow up or support groups for our age who are either pregnant or young mothers 2. Did anyone talk to you at some point about how to prepare for childbirth and infant care (if yes, who and what did they tell you exactly?) 3. Please tell me your views regarding the information you received from the clinic you have been or are attending   Probe:   - 1. Information on pregnancy   2. Information on delivery   3. Information on Infant care   4. Information on emotional, physical and social wellbeing  1. Please describe to me how you have been feeling through your pregnancy and motherhood   Probe:   - Negative feelings- anger, depression, worthlessness, hopelessness, disappointed - Positive feelings: Happiness, peace, hopeful, excited, purposeful, settled  1. Please tell me what you think are the causes or contributing factors to how you have been feeling? tell me more   Probe:   - Economic stress, - Emotional stress, - Physical stress - Family issues, - School-related problems, - Conflicts with the baby’s father  1. I would like you to tell me how you cope when you face these challenges mentioned above.   Probe:   - - What do you usually do when you feel low?   - Who do you talk to?   - Where do you or would you get support?  1. For first time pregnant ONLY: Please tell me the changes you expect to see in the next 1 year with this child once he/she is born?   Probe:   - - Economic changes   - School attendance/performance   - Emotional and social changes   - Physical changes  1. How would you describe how confident you are in the upcoming motherhood roles 2. Delivery process 3. Breastfeeding practice 4. Your nutrition 5. Physical changes in your body 6. Infant care process 7. For mothers ONLY: How has your motherhood journey been?  - In terms of how you generally feel about your situation everyday - In terms of how you feel emotionally - In terms of your economic state - In terms of adjusting to a new life (school, work) - In terms of your feelings towards the child/children - In terms of your thoughts about the future  1. Please tell me the kind of support you usually get to help you take care of yourself and your children (*probe emotional and financial*). And from whom you usually get that support from   **Views on potential support activities for pregnant and adolescent mothers**   1. Please describe the support available for pregnant and adolescent mothers in the informal settlements (slums where you live) and who offers this support   Probe:   - 1. Health-related support   2. Economic support   3. Nutritional support   4. Emotional support   5. Social support  1. In your opinion, what would adolescent mothers in Nairobi slums need most to make them feel really supported during their pregnancy and (coming) motherhood?   Probe:   - Information on birth preparedness and complication readiness information to cope with pregnancy-related stress). *If yes, Why? If no Why?* - Problem-solving skills (e.g. how to handle being chased away from home or from school because of the pregnancy). *If yes, Why? If no Why?* - Confidence- building on matters of motherhood (breastfeeding, infant feeding and nurture, maternal nutrition). *If yes, Why? If no Why?* - Any other?  1. Looking at your own case, what would you say you need or would have needed the most to make your journey a very positive and fulfilling one? *Probe each time further to get details*. |
| --- |
